# Transcriptome Differences Suggest Novel Mechanisms for Intrauterine Growth Restriction Mediated Dysfunction in Small Intestine of Neonatal Piglets

**DOI:** 10.3389/fphys.2020.00561

**Published:** 2020-06-23

**Authors:** Shimeng Huang, Zhenhua Wu, Xiongkun Yuan, Na Li, Tiantian Li, Junjun Wang, Crystal L. Levesque, Cuiping Feng

**Affiliations:** ^1^Department of Obstetrics and Gynecology, China-Japan Friendship Hospital, Beijing, China; ^2^State Key Laboratory of Animal Nutrition, College of Animal Science and Technology, China Agricultural University, Beijing, China; ^3^Department of Animal Sciences, South Dakota State University, Brookings, SD, United States

**Keywords:** intrauterine growth restriction, intestinal dysfunction, inflammatory, lipid metabolism, neonatal piglets, transcriptomics

## Abstract

Impaired intestinal function is frequently detected in newborns with intrauterine growth restriction (IUGR), whereas the mechanism between transcriptome profiles and small intestinal dysfunction is still unclear. Therefore, this study was conducted by using IUGR neonatal piglets to uncover the mechanism underlying intestinal dysfunction. Neonatal piglets with IUGR and normal birth weight (NBW) were sacrificed at birth. Transcriptomic sequencing was performed on jejunum samples and generated 18,997 and 17,531 genes in NBW and IUGR groups, respectively. A total of 10 differentially expressed genes (DEGs) were identified; of note, only seven were mapped to the genome reference database, with two up-regulated (*HSF4* and *NR1H4*; heat shock transcription factor 4 and nuclear receptor subfamily 1 group H member 4, respectively) and five down-regulated (*SLC35C1*, *BTNL3*, *BPI*, *NLRP6*, and *SLC5A8*; Solute carrier family 35 member C1, butyrophilin like 3, bactericidal permeability increasing protein, NLR family pyrin domain containing 6, and solute carrier family 5 member 8, respectively). Combining an enrichment analysis and reverse transcriptase–quantitative polymerase chain reaction validation of DEGs, our results proved the lipid metabolism disorder, intestinal dysfunction, and inflammatory response in IUGR piglets. Here, IUGR piglets presented lower concentration of glucose and triglyceride and higher concentration of total cholesterol and low-density lipoprotein cholesterol in plasma, compared with NBW piglets. Histological analysis revealed decreased mucins and increased apoptosis in both jejunum and ileum for IUGR piglets. Collectively, we found that IUGR induced intestinal dysfunction by altering lipid metabolism, intestinal barrier, and inflammatory response in neonatal piglets at birth, which provides new insights into the prevention and treatment of IUGR that protects against metabolic disorders and inflammatory-related diseases.

## Introduction

Intrauterine growth restriction (IUGR) refers to impaired growth and development of the mammalian embryo/fetus or its organs during pregnancy, which is defined by fetal or birth weight more than 2 standard deviations (SDs) below the mean body weight for gestational age (Wu et al., 2008; Wang et al., 2010). As a crucial health problem, IUGR not only causes neonatal mortality and morbidity in 5–10% human neonates worldwide, but also leads to adverse effects on postnatal growth and threatens long-term health (Widdowson, 1971; Sangild et al., 2006; Tomasz et al., 2007). Besides, IUGR is a multifactor disease associated with inadequate nutrient intake, environmental stress, and malfunction of the placenta or uterus (McMillen and Robinson, 2005).

Because of the severe prevalence of IUGR across other mammalian species (Wu et al., 2006) and the similarity of gastrointestinal tract with respect to structure, development, and metabolism (Wang et al., 2008; Ferenc et al., 2014), piglets are often applied to the study of the IUGR syndrome of human neonates. Previous studies have reported that IUGR hinders the development of piglet small intestine (SI) by changing the expression of proteins and genes related to cellular signaling, redox balance, protein synthesis, and lipid metabolism (Wu et al., 2006; Wang et al., 2008, 2010, 2018; Li et al., 2017; Huang et al., 2020). Small intestine (SI) is a crucial organ for food digestion and nutrient absorption, as well as a natural defensive barrier against endogenous pathogens and dietary contaminations (Hsueh et al., 2003; Sangild et al., 2006). The abnormal alterations during SI development may trigger feeding intolerance, fat absorption disorder, and digestive disease in newborns (Bernstein et al., 2000; Lee et al., 2001). Furthermore, certain studies detected morphological changes of SI in postnatal piglets, including intestinal length, villous height, and crypt depth, as well as decreased body weight, which may be associated with the symptoms of IUGR (Xu et al., 1994; Baserga et al., 2004). There are several studies focused on liver and islet transcriptome diversity between growing pigs and rat, respectively, but no study has compared differences in the SI transcriptome between the normal birth weight (NBW) piglets and IUGR piglets at birth.

Analysis on differences in the transcriptome between NBW and IUGR neonatal piglets at birth can help us better understand the underlying mechanism of IUGR-induced intestinal dysfunction, metabolic disorders, and inflammatory development and seek appropriate strategies for the prevention and treatment of the disease. Therefore, this study is conducted to (1) perform RNA-seq to identify the genes responsible for the intestinal alteration; (2) conduct reverse transcriptase–quantitative polymerase chain reaction (RT-qPCR) validation and histological analysis on jejunum to detect differentially expressed genes (DEGs) expression levels and mucosal barrier alterations between groups, respectively, and verify transcriptomic findings.

## Materials and Methods

### Ethics Statement

All experiments were carried out with the approval of China Agricultural University Animal Care and Use Committee (CAU20170114-1, Beijing, China).

### Animal Experiment Design and Sample Collection

In the current study, a total of nine multiparous sows (Yorkshire, 2–4 parities) were selected and raised in a commercial pig breeding farm in Sichuan province, China. During the experimental period, the selected sows were fed the same commercial feed and had free access to clean water. Porcine neonates (Landrace × Yorkshire) were spontaneously delivered from sows, and their body weights were recorded at birth. IUGR was identified when the piglet’s body weight was 2 SDs below the mean body weight of the total population. Then, one IUGR with the mean body weight of 0.92 ± 0.04 kg and one NBW with the mean body weight of 1.44 ± 0.05 kg piglets were selected in each of nine litters.

For sampling, a total of 18 male piglets (nine IUGR and nine NBW) were weighed and then killed by a jugular puncture after anesthesia without suckling, as we described previously (Wang et al., 2008, 2018). Blood samples were drawn into heparin-treated tubes via anterior vena cava puncture, and piglets were killed by jugular exsanguination. Plasma was obtained by centrifugation (3,000 revolutions/min for 15 min at 4°C) and was then stored at −80°C. Tissues from all selected IUGR and NBW neonatal piglets were collected. The SI in the neonatal piglet was defined as the portion of the digestive tract from the pylorus of the stomach to the ileocecal valve, with the first 10-cm segment being duodenum. The jejunum and ileum are the distal two parts of the SI. The jejunum begins at the duodenojejunal flexure and the ileum ends at the ileocecal junction. The subsequent 40 and 60% of the SI length below the duodenum are the jejunum and the ileum, respectively (Wang et al., 2008, 2018). The intestine was dissected free of the mesentery and placed on a chilled stainless steel plate. Segments (5 and 10 cm in length) were obtained, respectively, from the midjejunum and midileum (Wang et al., 2008; Yi et al., 2018). Meanwhile, the segments of 10 cm were opened longitudinally and carefully flushed with ice-cold phosphate-buffered saline. Then, the mucosa was collected by scraping using a microscope slide at 4°C and rapidly stored in liquid nitrogen of −80°C until further analysis.

### RNA Extraction and Sequencing

All experiments were carried out with three biological replicates, and each biological replicate contained three piglets from the same group. The jejunum mucosa was collected randomly from three independent piglets at the same group and then pooled together for the following measurement of RNA extraction until further RNA-seq. For RNA extraction, samples were frozen directly in liquid nitrogen after harvest and then ground into powder.

Total RNA was extracted from intestinal sample using TRIzol reagent (Invitrogen, Carlsbad, CA, United States) according to the manufacturer’s instructions, and RNA-seq was performed by Majorbio BioTech Co., Shanghai, China. RNA purity and concentration were measured using a Nano Photometer^®^ spectrophotometer (IMPLEN, Los Angeles, CA, United States) and a Qubit^®^ RNA Assay Kit in a Qubit^®^ 2.0 Fluorometer (Life Technologies, Carlsbad, CA, United States). RNA integrity was assessed using an RNA Nano 6000 Assay Kit in a Bioanalyzer 2100 system (Agilent Technologies, Santa Clara, CA, United States). Only samples with RNA integrity number >8 were used for sequencing. Illumina HiSeq 2500 platform was applied to construct RNA libraries and generate reads of 125-bp long paired-end (Illumina, San Diego, CA).

### RNA-Seq Data Processing

Clean data were obtained by removing reads containing adapters and greater than 10% of poly(N) and low-quality reads (>50% of the bases had Phred quality scores <10) from the raw data. All the downstream analyses were based on the high-quality clean data. *Sus scrofa* reference genome and gene model annotation files were downloaded from the NCBI database. Index of the reference genome was built using Bowtie v2.0.6 (Broad Institute, Cambridge, MA, United States), and paired-end clean reads were aligned to the reference genome using TopHat v2.0.14. Cufflinks v2.2.1 was applied to assemble mapped reads from each library and identify mRNA transcripts from the TopHat2 alignment results using the reference annotation based on transcript assembly method (Trapnell et al., 2012).

### Differential Expression Analysis

Cuffdiff v2.1.1 was used to calculate FPKM (fragment per kilo base of exon model per million mapped reads) scores for transcripts in each library (Trapnell et al., 2012). Differentially expressed genes were identified through pairwise comparisons between every two stages by DESeq2. Also, we analyzed the expression clustering by systematic analysis for all DEGs using the R package DESeq (Benjamini and Hochberg, 1995). A false discovery rate (FDR) was preset to a number no larger than 0.01, following the procedure of Benjamini-Hochberg (Calafat et al., 1998), which was used to determine the threshold of *P*-value in multiple tests of differential expression genes. The statistical significance of gene expression differences was evaluated using *P* < 0.05 and a fold change ≥ 1.5 as a threshold. In addition, we analyzed the expression clustering by systematic analysis for all DEGs using the Heatmaps software package in R (ggplot2 package). Ten DEGs with the most significant alteration were presented as a volcano plot. The log2-transformed fold changes are plotted for abscissa and the -log10-transformed Padjust for ordinate.

### GO and KEGG Pathway Analysis

Gene Ontology (GO) analysis of DEGs was carried out using Cytoscape software (version 3.4.0; Cytoscape Consortium, San Diego, CA, United States) with the BiNGO application. Kyoto Encyclopedia of Genes and Genomes (KEGG) analysis of DEGs was performed with KOBAS software using a hypergeometric test. GO terms and KEGG pathways with a *Q* <0.05 were considered significantly enriched.

### Validation of Gene Expressions by RT-qPCR

To validate the repeatability and reproducibility of DEGs obtained from RNA-seq, five DEGs were quantified by RT-qPCR. Primers were designed based on primer Premier 7.0 and synthesized by Shanghai Generay Biotech Co., Ltd. (Shanghai, China) which are shown in Supplementary Table 1. RNA extraction, cDNA synthesis, and RT-qPCR were conducted according to the method as described previously (Huang et al., 2020). Total RNAs from midjejunum and midileum segment were extracted using Trizol Reagent (Invitrogen) following the manufacturer’s protocol. The RT-qPCR was performed according to the SYBR Premix Ex Taq^TM^ II instructions (Takara, Tokyo, Japan). The reactions were performed on a LightCycler^®^ System (Roche, Mannheim, Germany) as follows: 95°C for 1 min, followed by 40 cycles of 95°C for 10 s, and 10 s at the annealing temperature (Tm). Melting curve analysis was performed from 65 to 95°C with increments of 0.5°C. The relative mRNA levels of target samples to that of control samples were calculated according to 2^–ΔΔCt^ method (Bustin et al., 2009), in which the difference in Ct values (ΔCt) between the target gene and the reference gene/internal control (β-actin) was calculated for normalization, and the ΔCt of the different samples was compared directly (ΔΔCt). Each RNA sample was analyzed in nine biological replicate. Data were expressed as least-square means ± standard error of the mean (SEM).

### Blood Biochemical Parameters Analysis

The concentrations of glucose, triglyceride (TG), total cholesterol (TC), high-density lipoprotein cholesterol (HDL-C), and low-density lipoprotein cholesterol (LDL-C) in plasma were assayed by Hitachi automatic biochemistry analyzer 7160 (Hitachi High-Tech Corporation, Tokyo, Japan) with Maccura chemical reagents (Maccura Biotechnology Co., Ltd., Chengdu, China).

### Validation of Intestinal Dysfunction by Histological Analysis

The midjejunum and midileum were collected, fixed in 4% paraformaldehyde (4°C), dehydrated in graded alcohol, and embedded in paraffin wax. Then, the embedded tissues were cut into 4-μm-thick sections and stained with Alcian blue (AB) and periodic acid–Schiff for morphological analysis. After deparaffinization and rehydration were performed, the slices were treated with AB G8X (Servicebio, G1049) for 5 min and washed with running tap water for 2 min. Afterward, slices were treated with periodic acid (Servicebio, G1049) for 5 min and subjected to Schiff’s staining (Servicebio, G1049) for 30 min in the dark. Afterward, the slices were stained with hematoxylin, dehydrated, mounted, and developed. The images were evaluated using the Image J software (US National Institutes of Health, Bethesda, MD, United States).

For gut apoptosis detection, it was detected by terminal deoxynucleotidyl transferase-mediated dUTP nick-end labeling (TUNEL) staining of sections of jejunal and ileal tissues using an *In Situ* Cell-Death Detection Kit (Roche, Basel, Switzerland) following the manufacturer’s instructions. Images were captured using an Olympus BS43 microscope (Olympus, Tokyo, Japan) and processed using DP73 version software (Olympus). The software was used to select the green fluorescent cell nuclei with the same label as the unified standard for judging all photopositive cells. The DAPI blue nuclei with the same label were selected as the total cells, and the TUNEL-positive cell number per field of intestinal epithelial cells was analyzed. Cell apoptosis was observed by green fluorescence microscopy (200 × magnification).

### Statistical Analysis

**FIGURE 1:**
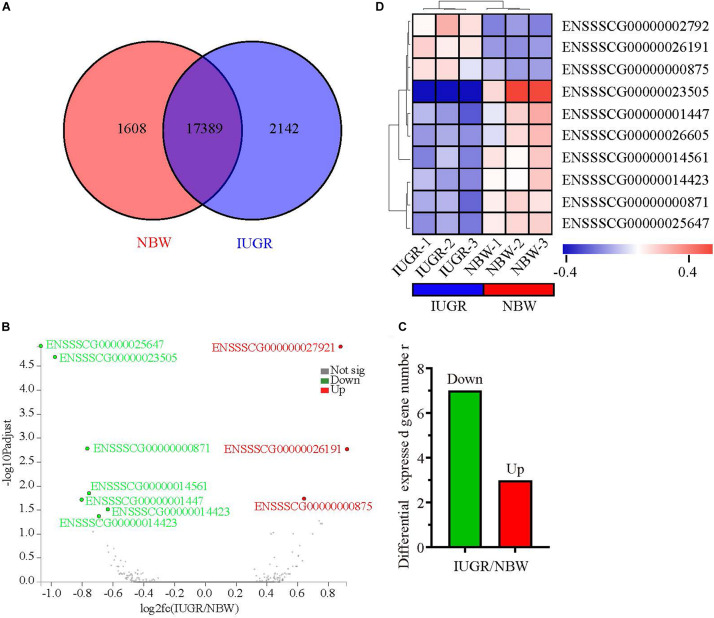
Genome-wide distribution of differentially expressed genes between NBW and IUGR piglets at birth. **(A)** Venn diagram of expressed genes between NBW and IUGR piglets. **(B)** Volcano plots of the most significantly altered genes. **(C)** Heatmap diagram of differentially expressed genes between NBW and IUGR piglets. **(D)** Distribution of up-regulated genes in NBW and IUGR piglets.

The minimum sample size of three samples per group (*n* = 3 in the study) was calculated using RNA-seq in the liver of adult pigs (Shen et al., 2018). Litter variation (*P* > 0.05) was tested using linear regression model (mixed effects). A logarithmic transformation was applied to the fold change of gene abundance in order to generate Figure 1B. Gene expression abundance of NBW sample at birth was used as the denominator, by which the gene expression abundance of all samples was compared. To compare the differences among the groups, one-way analysis of variance and Duncan *post-hoc* test for multiple comparisons were used for normally distributed data, whereas the Kruskal–Wallis test was used for non–normally distributed data. All analyses were performed using SAS 9.0 statistical software (SAS, Cary, NC, United States). Data are expressed as means with their SEM, and values of *P* < 0.05 were considered significant.

## Results

### Summary of Transcriptomic Data

A total of 247,167,220 raw reads were generated from RNA-seq. Subsequently, 23 million clean reads were obtained after the low-quality and adaptor sequences were filtered out. Approximately 80.58–81.99% of all reads were mapped to the reference genome, and 74.74–75.96% of all reads were unique and aligned to the University of California, Santa Cruz pig reference genome (*S. scrofa*) using the TopHat2 package (Table 1). It was considered to be an expressed transcript and included for subsequent analysis. Therefore, 18,997 and 19,531 known transcripts were identified as being expressed in NBW piglets and IUGR groups, respectively. To explore the global transcriptional changes, we identified a total of 17,389 transcripts coexpressed in both groups, whereas 1,608 and 2,142 genes were differentially expressed in the NBW and IUGR groups, respectively (Figure 1A). Based on the threshold of *P* < 0.05 and fold change ≥ 1.5, 10 DEGs with the most significant alteration were identified and presented as a volcano plot, with seven down-regulated and three up-regulated in the IUGR group (Figures 1B,C and Table 2). Interestingly, only seven genes were mapped to the pig genome database (PiGenome), with two up-regulated (*HSF4* and *NR1H4*) and five down-regulated (*SLC35C1*, *BTNL3*, *BPI*, *NLRP6*, and *SLC5A8*). The heatmap of the hierarchical clustering analysis revealed the significantly differential expressions between the two groups and indicated the high reproducibility of these DEGs (Figure 1D).

**TABLE 1:** Summary of transcriptomic results.

Items	NBW			IUGR		
		
	NBW-1	NBW-2	NBW-3	IUGR-1	IUGR-2	IUGR-3
Total reads	41,063,624	40,908,798	41,268,792	41,189,702	41,425,826	41,310,478
Clean reads	39,836,170	40,046,118	39,911,748	39,590,086	39,506,718	39,867,632
Valid ratio (reads)	0.99	0.99	0.99	0.99	0.99	0.99
Total mapped ratio (%)	81.99	81.86	81.62	81.24	80.58	81.95
Uniquely mapped ratio (%)	75.89	75.96	75.66	75.22	74.74	75.90
Q20 (%)	97.19	97.16	97.13	97.07	97.12	97.14
Q30 (%)	92.52	92.45	92.38	92.21	92.3	92.41
GC content (%)	50.57	50.32	51.07	51.02	52.3	50.97

*NBW, normal body weight; IUGR, intrauterine growth restriction.*

**TABLE 2:** Differentially expressed genes of IUGR piglets in relative to NBW piglets with a fold change ≥ 1.5 and *P* ≤ 0.05.

Ensembl gene ID	Gene name	Fold change	Log2 Fold change	*P*	Adjusted *P*	Regulation in IUGR piglets
ENSSSCG00000025647	*SLC35C1*	0.48	–1.07	1.92 × 10^9^	1.24 × 10^–5^	Down
ENSSSCG00000000875	*NR1H4*	1.55	0.63	9.94 × 10^6^	0.02	Up
ENSSSCG00000026191	*–*	1.89	0.92	6.62 × 10^7^	<0.01	Up
ENSSSCG00000014423	*–*	0.65	–0.63	2.21 × 10^5^	0.03	Down
ENSSSCG00000026605	*BPI*	0.62	–0.69	3.29 × 10^5^	0.04	Down
ENSSSCG00000001447	*BTNL3*	0.57	–0.80	1.19 × 10^5^	0.02	Down
ENSSSCG00000002792	*HSF4*	1.84	0.88	6.37 × 10^10^	8.20 × 10^–6^	Up
ENSSSCG00000014561	*NLRP6*	0.59	–0.76	6.76 × 10^6^	0.02	Down
ENSSSCG00000000871	*SLC5A8*	0.59	–0.77	6.28 × 10^7^	<0.01	Down
ENSSSCG00000023505	*–*	0.51	–0.98	4.87 × 10^9^	2.09 × 10^–5^	Down

*NBW, normal body weight; IUGR, intrauterine growth restriction.*

**FIGURE 2:**
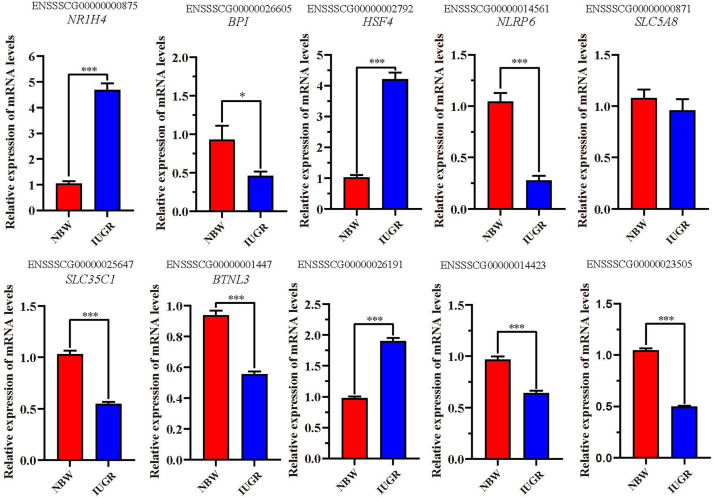
Reverse transcriptase–quantitative polymerase chain reaction validation of the selected genes. Data are shown as mean ± SEM. ^∗^*P* < 0.05, ^∗∗∗^*P* < 0.001. *n* = 9 per group. NBW, normal body weight; IUGR, intrauterine growth restriction.

A total of 10 genes including *NR1H4*, *BPI*, *HSF4*, *NLRP6*, *SLC5A8*, *SLC35C1*, *BTNL3*, ENSSSCG00000026191, ENSSSCG00000014423, and ENSSSCG00000023505 were selected for RT-qPCR analysis. As shown in Figure 2, no significant change was observed for *SLC5A8* mRNA levels between NBW and IUGR groups. Conversely, the mRNA levels of *NR1H4*, *HSF4*, and ENSSSCG00000026191 were significant up-regulated (*P* < 0.05) in the IUGR group. Meanwhile, the gene expressions of *BPI*, *NLRP6*, *SLC35C1*, *BTNL3*, ENSSSCG00000014423, and ENSSSCG00000023505 were lower (*P* < 0.05) in the IUGR piglets compared to NBW piglets.

### Functional Enrichment Analysis of DEGs

**FIGURE 3:**
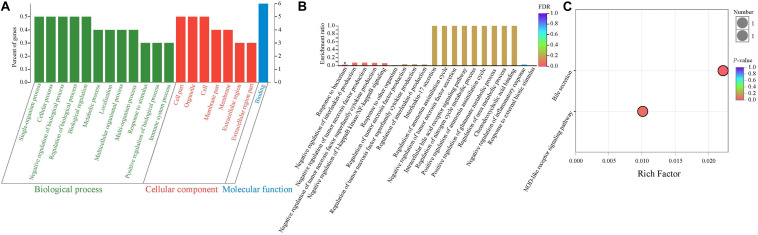
Functional enrichment analysis of differentially expressed genes between NBW and IUGR piglets. **(A)** Gene Ontology categories enriched for differentially expressed genes. The abscissa represents the secondary classification terms of GO, the left ordinate represents the percent of genes enriched in the secondary classification, and the right ordinate represents the number of genes. These three colors represent three categories: green represents the biological process, red represents the cellular component, and blue represents the molecular function. **(B)** Gene Ontology enrichment analysis enriched for differentially expressed genes. Significantly enriched GO terms were selected based on a FDR < 0.05. **(C)** KEGG pathway enrichment analysis of differentially expressed genes.

In order to determine the potential functions of these 10 DEGs, the GO enrichment analysis was performed. Therefore, our results showed that more genes were involved in biological processes, followed by cellular components and molecular functions (Figure 3A). As presented in Figure 3B, most GO terms were related to two main classes of biological process: inflammatory and immune activity (such as negative regulation of interleukin 6 production, negative regulation of tumor necrosis factor production, negative regulation of IκB jinase/nuclear factor κB signaling, and negative regulation of inflammatory response) and metabolism (such as regulation of ammonia assimilation cycle, intracellular bile acid receptor signaling pathway, regulation of nitrogen cycle metabolic process, and positive regulation of glutamate metabolic process). In addition, the enrichment ratio of GO terms related to metabolism was higher than those related to inflammatory and immune activity. Interestingly, our results showed that the significantly overrepresented GO term was the response to the bacterium (*P* < 0.05).

Next, the KEGG pathway enrichment analysis was performed. As shown in Figure 3C, the DEGs were significantly enriched in the bile secretion pathway and NOD-like receptor signaling pathway (*P* < 0.05), which are related to metabolism and immune and inflammatory activity, respectively.

### Blood Chemical Parameters Analysis

In the analysis of lipid metabolites in plasma, IUGR group exhibited lower concentrations of glucose (*P* < 0.05) and TG (*P* < 0.01) and higher concentrations of TC (*P* < 0.05), HDL-C, and LDL-C, *P* < 0.05) compared with the normal group. Details are presented in Table 3.

**TABLE 3:** Concentrations of lipid metabolites in plasma between NBW and IUGR neonatal piglets.

Items	NBW	IUGR	*P*
Glucose (mmol/L)	4.67 ± 1.45	3.04 ± 0.40	0.0392
TG (mmol/L)	1.51 ± 0.23	1.11 ± 0.04	<0.001
TC (mmol/L)	0.16 ± 0.03	0.27 ± 0.03	0.0018
HDL-C (mmol/L)	0.43 ± 0.10	0.49 ± 0.08	0.2728
LDL-C (mmol/L)	0.40 ± 0.06	0.54 ± 0.11	0.0187

*Values are presented as means ± SEM. NBW, normal body weight; IUGR, intrauterine growth restriction; TG, triglyceride; TC, total cholesterol; HDL-C, high-density lipoprotein cholesterol; LDL-C, low-density lipoprotein cholesterol. n = 9 per group. NBW, normal body weight; IUGR, intrauterine growth restriction.*

### Histological Analysis

**FIGURE 4:**
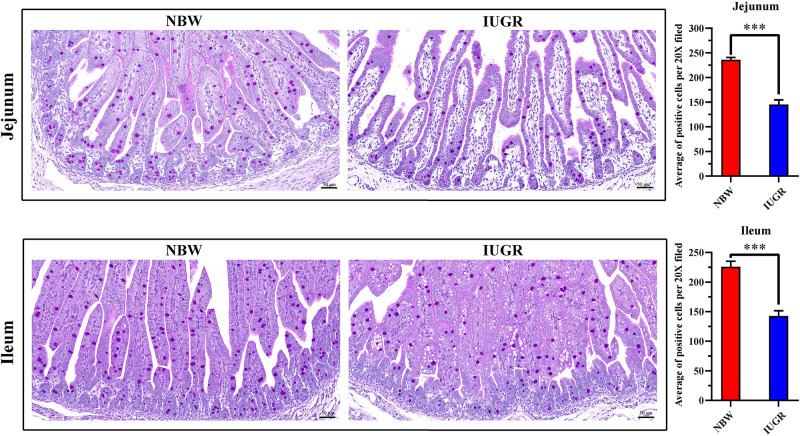
Periodic acid–Schiff staining on jejunum and ileum in NBW and IUGR piglets. Data are shown as mean ± SEM. ^∗∗∗^*P* < 0.001. *n* = 3 per group. NBW, normal body weight; IUGR, intrauterine growth restriction. Bar = 50 μm.

**FIGURE 5:**
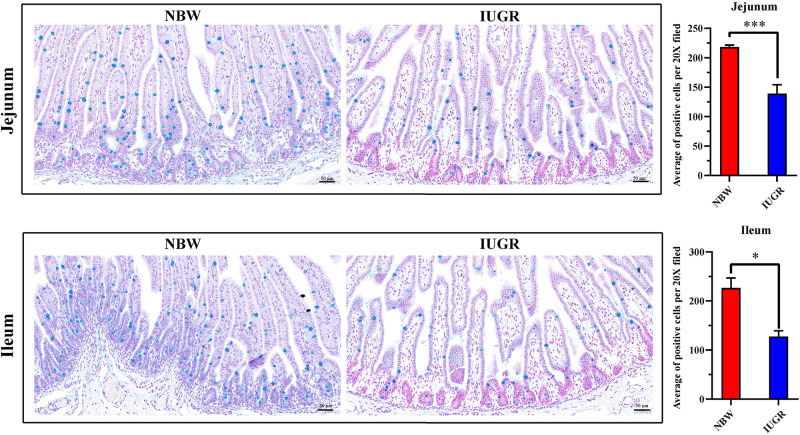
Alcian blue staining on jejunum and ileum in NBW and IUGR piglets. Data are shown as mean ± SEM. ^∗^*P* < 0.05, ^∗∗∗^*P* < 0.001. *n* = 3 per group. NBW, normal body weight; IUGR, intrauterine growth restriction. Bar = 50 μm.

**FIGURE 6:**
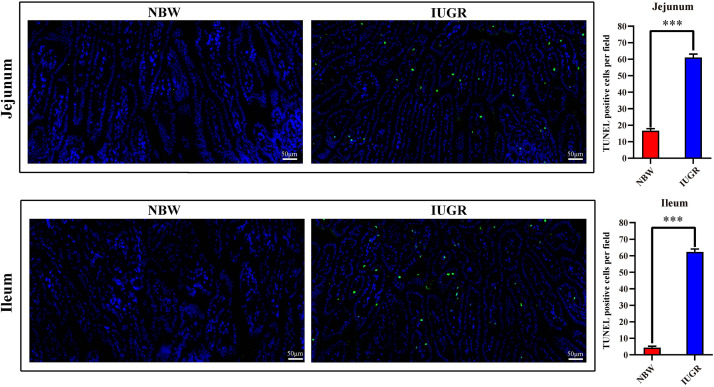
Apoptosis analysis on jejunum and ileum by TUNEL in NBW and IUGR piglets. Data are shown as mean ± SEM. ^∗∗∗^*P* < 0.001. *n* = 3 per group. TUNEL, terminal deoxynucleotidyl transferase dUTP nick end labeling; NBW, normal body weight; IUGR, intrauterine growth restriction. Bar = 50 μm.

Periodic acid–Schiff and AB staining analysis revealed that the IUGR group presented fewer positive cells in both jejunum (*P* < 0.005) and ileum (*P* < 0.05) compared with the NBW group (Figures 4, 5). Terminal deoxynucleotidyl transferase-mediated dUTP nick-end labeling histological analysis revealed that IUGR group presented more apoptotic cells in both jejunum (*P* < 0.001) and ileum (*P* < 0.05) compared with the NBW group (Figure 6).

### Expression of Genes Related to Intestinal Dysfunction Analysis

**FIGURE 7:**
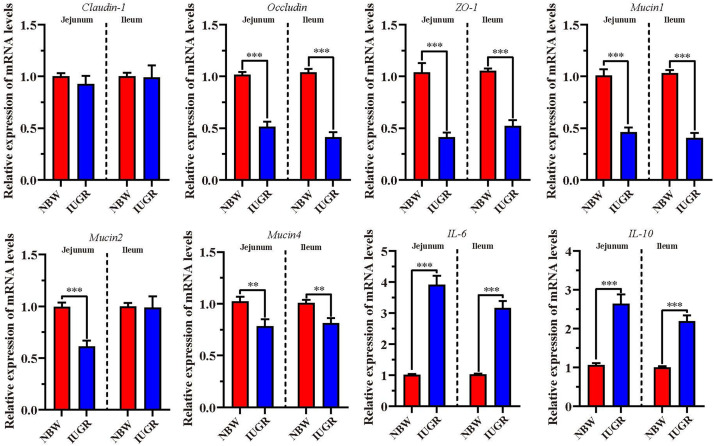
Analysis of genes involved in intestinal barrier and proinflammatory cytokines. Data are shown as mean ± SEM. ^∗∗^*P* < 0.01, ^∗∗∗^*P* < 0.001. *n* = 9 per group. NBW, normal body weight; IUGR, intrauterine growth restriction.

To validate intestinal dysfunction results, gene mRNA expressions of interest were detected to perform RT-qPCR in both jejunum and ileum. As shown in Figure 7, the mRNA levels of *occludin*, zonula occludens-1 (*ZO-1*), *Mucin1*, and *Mucin4* were significantly down-regulated (*P* < 0.01) in jejunum and ileum tissues from IUGR piglets. Besides, *Mucin2* was remarkably down-regulated (*P* < 0.001) in the jejunum from IUGR group. Conversely, we observed that the expression of proinflammatory genes interleukin 6 (*IL-6*) and interleukin-10 (*IL-10*) was significantly increased (*P* < 0.001) in jejunum and ileum from IUGR piglets.

## Discussion

Intrauterine growth restriction is a global disease associated with high mortality and morbidity in both human and animals, especially in pig neonates, which triggers great financial loss in the swine industry. Previous studies have detected that IUGR affects the development of infant intestine, leading to intestinal dysfunction and metabolic disorders (Wang et al., 2008, 2018; Li et al., 2017; Huang et al., 2020). Therefore, the current study was performed with the purpose to understand the mechanism of IUGR on neonatal intestine at birth.

By utilizing RNA-seq, we identified 10 significantly altered genes between the groups, which are involved in the GO terms and pathways related to metabolism and inflammatory and immune activity. Among the 10 DEGs, only seven were mapped to the genome reference database, with two up-regulated (*HSF4* and *NR1H4*) and five down-regulated (*SLC35C1*, *BTNL3*, *BPI*, *NLRP6*, and *SLC5A8*).

As a member of the NR-family, *NR1H4* (or FXR) is expressed predominantly in the intestine and plays a major role in maintaining bile acid (BA) homeostasis (Cariou and Staels, 2007; Fiorucci et al., 2007; Zhang and Edwards, 2008), which is important for the regulation of lipid, glucose, and energy metabolism (Lee et al., 2006; Cariou and Staels, 2007; Staels and Kuipers, 2007; Zhang and Edwards, 2008). In addition, the effect of BA to modulate TG metabolism is primarily dependent on the activation of FXR, which changes the transcription of the genes related to fatty acid and TG synthesis and lipoprotein metabolism (Sirvent et al., 2004; Watanabe et al., 2004). According to our previous studies, low birth weight or IUGR may damage lipid metabolism and intestinal barrier and aggravate malnutrition and metabolic dysfunction in IUGR piglets during early life (Wang et al., 2008; Huang et al., 2019, 2020). In the present study, our result has shown that the concentrations of TC and LDL-C were increased, and the levels of glucose and TG were decreased in IUGR piglets, compared with the NBW piglets. *NR1H4* was demonstrated to reduce plasma glucose and TC in FXR-null mice (Lambert et al., 2003; Ma et al., 2006; Zhang et al., 2006). Hence, in the current study, we observed that IUGR group presented significantly higher expression of *NR1H4* and lower concentration of glucose and TG compared with the NBW group, suggesting that IUGR affects intestinal homeostasis by regulating lipid metabolism and explaining the IUGR symptoms such as metabolic disorder and low birth weight in neonates.

In addition, we have observed the significant alteration of immunity and inflammation-related genes between the NBW and IUGR piglets. The Nod-like receptor, NLRP6, is recognized as inflammasome that is highly expressed in small and large intestine (Deo and Bandiera, 2008; Hofmann and Hagey, 2008; Russell, 2009). It has been demonstrated to promote intestinal homeostasis and prevent inflammatory disease such as colitis and colitis associated to tumorigenesis (D’Inca et al., 2011). Consistent with our results, expression of *NLRP6*, *occludin*, *ZO-1*, *Mucin1*, and *Mucin4* was shown to decrease upon IUGR in the SI, suggestive of intestinal dysfunction and inflammatory disease in IUGR piglets at birth. Chen et al. (2011) created a chemically induced colitis model in mice and found that the deficiency of *NLRP6* increased the susceptibility of injury and inflammation within the colon. Additionally, Al-Azemi et al. (2017) observed a decrease of anti-inflammatory cytokines in IUGR group compared with the normal controls, and D’Inca et al. (2011) detected that the expression of proinflammatory cytokine *IL-6* was modified in the intestine of IUGR piglets at birth, indicating the involvement of inflammatory activity in the mechanism of IUGR. Thus, *NLRP6*, *IL-6*, and *IL-10* exhibited significantly decreased expression level in IUGR group compared with the NBW group in our study. Because the intestinal function is closely linked to proinflammatory cytokines and inflammasome activation, the beneficial effects of *NLRP6* on intestinal homeostasis and inflammatory disease may add value to the use of *NLRP6* as a therapeutic target for dysregulation of inflammasome activation-associated intestine diseases.

BPI, an important antimicrobial polypeptide (Canny and Levy, 2008), plays a cardinal role in hindering the invasion of Gram-negative bacteria and inflammatory activity (Weiss et al., 1978). According to previous studies, BPI protein helps the host body in fighting with gram-negative bacteria by targeting its opsonization, by neutralizing LPS-mediated inflammatory responses, and through microbicidal activity (Canny and Levy, 2008; Palmer et al., 2011). We have shown that expression of *BPI* gene was significantly lower in IUGR piglets compared to NBW piglets, which we confirmed with RNA-seq and RT-qPCR analysis; similar results were reported in LBW newborns compared to NBW newborns (Singh et al., 2013). *BPI* dysregulation in intestinal epithelium is conceded to be associated with various inflammatory diseases, such as Crohn disease, ulcerative colitis, and infectious enteritis (Balakrishnan and Chakravortty, 2017). In agreement with our findings, the down-regulation of genes (*BPI*, *occludin*, *ZO-1*, and *Mucin1*) and up-regulation of genes (*IL-6* and *IL-10*) detected in IUGR piglets are suggestive of various inflammatory diseases and intestinal epithelium dysregulation in IUGR piglets.

Besides, the expression levels of *BTNL3*, *SLC5A8*, and *SLC31C1* were also altered in IUGR piglets. First, the butyrophilin-like (*BTNL*) genes are part of the immunoglobulin superfamily (Blazquez et al., 2018). Consistent with the recent studies, the role of *BTNL3* in the homing and maintenance of a semi-activated state on Vγ4 + γδ T cells in the human gut might be relevant for the onset of gut autoimmune diseases such as ulcerative colitis and inflammatory bowel disease (Lebrero-Fernandez et al., 2016; Blazquez et al., 2018). In this study, a down-regulation of *BTNL3* mRNA expression levels was observed in IUGR piglets at birth. Recent studies have shown the expression of *BTNL3* in tumors and its correlation with patient prognosis, immune response, and inflammatory bowel disease (Blazquez et al., 2018). Compared to NBW piglets at birth, similar results showed that the modulation of *BTNL3* seems of interest in the impaired intestinal barrier and inflammatory response in IUGR piglets. Secondly, SLC5A8 is regarded as a Na^+^-coupled high-affinity transporter for short-chain fatty acid, which is a vital factor for mucosal immune system (Gurav et al., 2015). Consistent with previous studies (Dong et al., 2014; Ferenc et al., 2014; Shalom-Paz et al., 2017), all these findings demonstrate that IUGR disturbs intestinal homeostasis by regulating the genes responsible for immune and inflammatory system, which predisposes the neonates to pathogens and increases the risk of mortality and morbidity. Finally, SLC35C1, also known as GDP-fucose transporter 1, is a member of the solute carrier (SLC) group protein (Fredriksson et al., 2008; Deng et al., 2020). In the previous study, *SLC35C1* is a negative regulator of the Wnt signaling pathway in gastrointestinal cancer and colon cancer (Petretti et al., 2000; Carvalho et al., 2010; Deng et al., 2020). Down-regulation of *SLC35C1* in colon cancer induces Wnt pathway activity, whose overactivation has been proved to be a hallmark of colon cancer (Deng et al., 2020). Interestingly, this group found that *SLC35C1* mRNA expression was decreased in IUGR piglets. This study also offers the potential to explore drugs that can restore *SLC35C1* to prevent intestinal diseases.

Additionally, heat shock transcription factors (HSFs) are highly conserved and are required for normal cell growth and development in addition to their central importance in stress adaptation, survival, and disease (Morimoto, 1993, 1998). HSF4 is a member of the heat shock transcription factor family that contains conserved DNA binding and trimerization domains (Morimoto, 1993; Nakai et al., 1997). Recent studies showed that HSF4 is expressed in many tissues and is required for mammalian cellular proliferation and differentiation (Min et al., 2004; Mitsuaki et al., 2004; Gao et al., 2017). Notably, Jin et al. (2012) observed HSF4 is positively involved in tumorigenesis that is induced following disruption of the *p*53 gene. Here, our analysis of RNA-seq and RT-qPCR showed that *HSF4* mRNA and cell apoptosis are up-regulated in IUGR group, revealing the existence of an inverse relationship between *HSF4* and IUGR.

As noted previously, our data further suggest that the alteration of SI transcriptional profiles is critical for this intestinal dysfunction and metabolic disorders in IUGR piglets at birth. Moreover, histological analysis revealed that mucosal epithelial cells were damaged. Here, our results showed that IUGR piglets presented fewer positive cells in both jejunum and ileum, compared with the NBW group. Mucus covers the intestinal surface and serves as the first line of innate defense, with mucins forming the basic skeleton. Alteration of mucins expression is considered to be related to inflammatory bowel diseases and colorectal cancer (Andrianifahanana et al., 2006). A lack of mucins has been observed in a model of mucosal injury (Lin et al., 2005), as well as in neonates with necrotizing enterocolitis (Mattar et al., 2003; Vieten et al., 2005), which is a disease of increased risk for IUGR neonates. Interestingly, consistent with previous studies (Canny and Levy, 2008; Wlodarska et al., 2014; Lebrero-Fernandez et al., 2016; Seregin et al., 2017) and our findings, expression of *NLRP6*, *BPI*, *BTNL3*, *occludin*, *ZO-1*, *Mucin1*, and *Mucin4* affects mucus secretion and intestinal barrier and contributes to intestinal homeostasis. Therefore, in the histological analysis on jejunum and ileum, we observed significantly increased apoptosis in IUGR group compared with the NBW group, indicating impairment of the intestinal mucosa in IUGR, which may explain the intestinal dysfunction and related syndromes in IUGR neonates.

## Conclusion

The present study uncovers that IUGR modulates lipid metabolism, intestinal barrier, and inflammatory activity, as well as impairs the intestinal mucosa in neonatal piglets. These findings may elucidate the primary mechanisms responsible for impaired growth in IUGR neonates and aid in the development of new nutritional intervention strategies.

## Data Availability Statement

The datasets generated for this study can be found in the NCBI BioProject accession PRJNA598528 (https://www.ncbi.nlm.nih.gov/bioproject/PRJNA598528).

## Ethics Statement

The animal study was reviewed and approved by the China Agricultural University Animal Care and Use Committee. Written informed consent was obtained from the owners for the participation of their animals in this study.

## Author Contributions

CF, JW, and SH designed the experiments. SH, NL, TL, and ZW conducted the experiment, performed the analysis of samples, and analyzed the data. SH, NL, XY, and ZW carried out the experiments and collected the samples. SH, CL, CF, and JW wrote the manuscript. All authors read and approved the final manuscript.

## Conflict of Interest

The authors declare that the research was conducted in the absence of any commercial or financial relationships that could be construed as a potential conflict of interest.

## Abbreviations

BPI: Bactericidal Permeability Increasing Protein
HSFs: Heat-Shock Transcription Factors
IUGR: Intrauterine Growth Restriction
NBW: Normal Birth Weight
NLRP6: NLR Family Pyrin Domain Containing 6
NR1H4: Nuclear Receptor Subfamily 1 Group H Member 4
DEGs: Differentially Expressed Genes
SI: Small Intestine
SD: Standard Deviation
SLC35C1: Solute Carrier Family 35 Member C1
SLC5A8: Solute Carrier Family 5 Member 8
SEM: Standard Error of the Mean
RABT: Reference Annotation Based On Transcript
FPKM: Fragment Per Kilo Base Of Exon Model Per Million Mapped Reads
GO: Gene Ontology
KEGG: Kyoto Encyclopedia of Genes and Genomes
AB: Alcian Blue
TG: Triglyceride
TC: Total Cholesterol
Tm: Annealing Temperature
TUNEL: Terminal Deoxynucleotidyl Transferase-Mediated Dutp Nick-End-Labeling
HDL-C: High Density Lipoprotein Cholesterol
LDL-C: Low Density Lipoprotein Cholesterol
IL-6: Interleukin-6
IL-10: Interleukin-10
FDR: False Discovery Rate
NCBI: National Center for Biotechnology Information
ZO-1: Zonula occludens-1.
