# Supplementary material for: Transcriptome Differences Suggest Novel Mechanisms for Intrauterine Growth Restriction Mediated Dysfunction in Small Intestine of Neonatal Piglets
Source: Front Physiol. 2020 Jun 23;11:561. doi: 10.3389/fphys.2020.00561 (PMC7324767; doi:10.3389/fphys.2020.00561)
Supplement: Supplementary file 1 [file Table_1.docx]

**Supplementary Table 1. The sequences of primers used in RT- PCR assays.**

| **Gene ID** | **Genes** | **Forward** | **Reverse** |
| --- | --- | --- | --- |
| ENSSSCG00000007585 | *β-Actin* | TGGAATCCTGTGGCATCCATGAAAC | TAAAACGCAGCTCAGTAACAGTCCG |
| ENSSSCG00000000875 | *NR1H4* | AAGGACCGAGAGGCAGTAGAGAAG | TCTGCGTGGTGATGGTTGAATGTC |
| ENSSSCG00000026605 | *BPI* | TTGAACAAGCTGCTCCTGGAACTG | AGGTTGGAGAGCTGGATGTAGGC |
| ENSSSCG00000002792 | *HSF4* | TGCTGCCTCCAATGCTGCTTC | GCTCCATGTCCAAGTCCATCAGAG |
| ENSSSCG00000014561 | *NLRP6* | TCCGTGTCAGCGTTCAAGAAGAAG | GAGCGAGCATTCCTCTCCTTCAC |
| ENSSSCG00000000871 | *SLC5A8* | CCTTAGCAGCAGTGACTGTGGAAG | TGATGCCAGCGCAGCCATTC |
| ENSSSCG00000025647 | *SLC35C1* | CTCGCTCACCACCGTCTTCAATG | GCCAGAAGCCGCCGATGATG |
| ENSSSCG00000001447 | *BTNL3* | CCAAACCCTCAGAACCTGTGTACG | ACTCTGCTCGCCCTTTGAAATCG |
| ENSSSCG00000026191 | - | GGATGTGACAAGCAGGCAAGGAG | GGTGGTCTGGAGTGGAGGGTATG |
| ENSSSCG00000014423 | - | CCACCACCATGAAGAGCATCACC | AGGCAACACTGGCACCTTTTCC |
| ENSSSCG00000023505 | - | ACCCTCTCATCCACCCAACTTCC | TATAGCCAGACTCACCTCCACAGC |
| ENSGT00940000155387 | *Claudin-1* | AGCTGTGCATGGCCTCTTGT | CCAATGTCAATGGCAACACCCT |
| ENSSSCG00000016965 | *Occludin* | CAGCCTCGGTACAGCAGCAAT | ATAGTGGTCAGGGTCCGTCCTC |
| ENSSSCP00000005227 | *ZO-1* | CGGAACTATGACCATCGCCTAC | CTTCGGGATGTTGTCTGGAGTC |
| ENSGT00710000106874 | *Mucin1* | AATGGCTCCTCGGTGCTACCTA | TGACTTGGCACTGAAGGCTGAG |
| ENSSSCT00000068079 | *Mucin2* | TGCTGACGAGTGGTTGGTGAATG | GATGAGGTGGCAGACAGGAGACA |
| ENSGT00730000110943 | *Mucin4* | TTCACTCCAACCATCCTTCCA | CTCGTTCCACTTGTCTGTTCC |
| ENSGT00390000000878 | *IL-6* | ACCACGGCCTTCCCTACTT | CACAACTCTTTTCTCATTTCCAC |
| ENSSSCT00005007269 | *IL-10* | GCCAAGCCTTGTCAGAGATGATCC | AGGCACTCTTCACCTCCTCCAC |
